# Relative vibrotactile spatial acuity of the torso

**DOI:** 10.1007/s00221-017-5073-6

**Published:** 2017-08-30

**Authors:** Ómar I. Jóhannesson, Rebekka Hoffmann, Vigdís Vala Valgeirsdóttir, Rúnar Unnþórsson, Alin Moldoveanu, Árni Kristjánsson

**Affiliations:** 1 Faculty of Psychology, School of Health Sciences, University of Iceland, Reykjavik, Iceland; 2 Faculty of Industrial Engineering, Mechanical Engineering and Computer Science, University of Iceland, Reykjavik, Iceland; 3 Faculty of Automatic Control and Computers, Polytechnic University of Bucharest, Bucharest, Romania

**Keywords:** Vibrotactile acuity, Vibrotactile accuracy, Discrimination of vibrotactile stimulation, Sensory substitution

## Abstract

While tactile acuity for pressure has been extensively investigated, far less is known about acuity for vibrotactile stimulation. Vibrotactile acuity is important however, as such stimulation is used in many applications, including sensory substitution devices. We tested discrimination of vibrotactile stimulation from eccentric rotating mass motors with in-plane vibration. In 3 experiments, we tested gradually decreasing center-to-center (c/c) distances from 30 mm (experiment 1) to 13 mm (experiment 3). Observers judged whether a second vibrating stimulator (‘tactor’) was to the left or right or in the same place as a first one that came on 250 ms before the onset of the second (with a 50-ms inter-stimulus interval). The results show that while accuracy tends to decrease the closer the tactors are, discrimination accuracy is still well above chance for the smallest distance, which places the threshold for vibrotactile stimulation well below 13 mm, which is lower than recent estimates. The results cast new light on vibrotactile sensitivity and can furthermore be of use in the design of devices that convey information through vibrotactile stimulation.

## Introduction

Tactile communication systems have been developed for a wide range of applications such as for navigation, alerting, and sensory substitution devices (SSDs) for people with visual and auditory impairments (Ranjbar et al. 2009; Yuan et al. 2005; Sampaio et al. 2001). The aim with SSDs is to assist sensory impaired people by partially restoring function with input from other senses. Touch or audition, for example, can be used to convey information that is otherwise not available, such as in vision loss (Maidenbaum et al. 2014; Jóhannesson et al. 2016).

SSDs have been developed for various body parts [see Kristjánsson et al. (2016), Jóhannesson et al. (2016), for reviews], such as the tongue (Tang and Beebe 2006; Chebat et al. 2011), hands (Ito et al. 2005), and fingertips (Koo et al. 2008). Applications that stimulate passive body parts (torso, arm) may be most practical however, since the tongue and hands should preferably be available for other use (Dakopoulos and Bourbakis 2010; Kristjánsson et al. 2016). Disadvantages of passive areas, such as the reduced sensitivity of hairy compared to glabrous skin (Bolanowski et al. 1994; Sofia and Jones 2013), as well as the reduced spatial acuity of these areas compared to active body parts (Weinstein 1968; Johnson and Phillips 1981), can be compensated for by the larger skin area that can be used for conveying information.

In a pioneering study Bach-Y-Rita et al. (1969) introduced a dental chair with a 20 × 20 array of tactors^1^ that translated visual information obtained by a camera into tactile stimulation on the participant’s back. Following extensive training (10–20 h, or even up to 150 h), participants were able to recognize objects, and some highly trained observers were able to report remarkable detail, such as whether people wore glasses or not. Since then, various mobile, tactile stimulation devices have been developed, in which vibration is presented to participants based on information from video cameras (McDaniel et al. 2008; Cosgun et al. 2014; Johnson and Higgins 2006) or a GPS receiver (Van Erp et al. 2005b). For instance, Van Erp et al. (2005b) developed a tactile belt with 8 tactors coding distance by vibration frequency and direction by vibration location. Collectively, these results suggest that tactile belts can be effective communication systems, especially for navigation.

The widespread use of mobile devices and wearable computers with limited screen space for visual information has led to increased interest in tactile communication systems. Vibrotactile equipment has become more accessible, affordable, as well as more sophisticated and less intrusive resulting in more effective and user-friendly designs (Jones and Sarter 2008).

Increased use of advanced tactile equipment highlights the importance of psychophysical investigations of basic mechanisms underlying human tactile perception. One of the main challenges in designing tactile displays is determining what type of information can be presented tactually and which parameters of stimulation can be used to effectively convey information. One issue involves empirical assessment of the maximal throughput of the skin. How many tactors can be arranged on the torso and how densely can they be placed before their loci become indistinguishable? Determining this threshold is important for the design of tactile displays since the more tactors can be placed within a defined area, the more information can be conveyed in space, which eliminates the need for encoding information by deploying the dimension of time. Psychophysical studies on tactile spatial acuity of different body sites can provide the required empirical basis for determining the optimal stimulation type for tactile displays.

### Tactile spatial acuity

In the nineteenth century Weber (1834) performed pioneering psychophysical research on relative spatial acuity of the skin, introducing two measurement methods that are still in use today. One is the two-point threshold (2PT), which is measured by presenting either one stimulus or two simultaneous stimuli while the distance between them is steadily decreased, to assess the threshold of when two stimuli are erroneously perceived as one. Another method is the point of localization (PL), which is assessed by successively presenting two tactile stimuli so that the second stimulus is either in the same location or increasingly distant from the first, in order to determine the threshold when two stimulation sites are correctly perceived as two. Weinstein (1968) used both methods to study the sensitivity of a large number of body parts and found that spatial tactile acuity with the two-point threshold was 2–4 times lower than with point localization although both thresholds were highly correlated over body parts. The lowest thresholds were found on the fingertips (2.5 and 1.5 mm, for PL and 2PT, respectively), whereas thresholds for the back were ca. 40 and 10 mm, respectively.

Although later studies have essentially confirmed Weinstein’s acuity map (Johnson and Phillips 1981; Stevens and Choo 1996), some argue that Weinstein’s methods underestimate the skin’s actual spatial acuity and have explored other assessment methods. Vierck and Jones (1969) measured discrimination of the size of plastic cylinders impressed on the forearm (on the palm side) concluding that the discrimination threshold is between 2 and 6 mm on the forearm. Jones and Vierck (1973) tested discrimination of the length of two straight lines impressed on the forearm, ranging in length from 1.6 to 127 mm. The average discrimination threshold was 21 mm or about two times lower than the 2PT threshold. This suggests that acuity can be higher than Weinstein’s results indicate, especially if patterns, rather than points are used for stimulation (Bach-y-Rita and Kercel 2003; Gibson 1962; Loomis et al. 2012).

### Vibrotactile spatial acuity

While tactile spatial acuity for pressure has been explored extensively, most applications nowadays use vibrotactile stimuli. All skin receptors, except the Ruffini endings, respond to vibrating stimuli but their sensitivity and frequency range differ substantially. The Pacinian corpuscles have the largest frequency range (5–1000 Hz) with peak sensitivity at 200 Hz. Furthermore, they do not respond to indentions of the skin (Gardner and Johnson 2013). A layer (e.g., a T-shirt) between the tactors and the skin can substantially reduce effects of lateral motion, stretching of the skin and the effects of edges and points which are the preferred stimuli of Meissner corpuscles, Ruffini endings and Merkel cell receptors, respectively. Generalizing threshold measures obtained with static pressure to vibratory stimuli may therefore be misleading. Furthermore, the size of the contact area may also influence the ability to localize vibrotactile stimuli, but this has apparently not been systematically investigated.

One way of determining the optimal spacing and number of vibrating tactors in tactile displays is to assess absolute spatial acuity. The method for measuring absolute spatial acuity [for example by Cholewiak et al. (2004), Lindeman and Yanagida (2003)] is to present one vibrotactile stimulus within a display of tactors with fixed distances and ask the participants to indicate the location of the activated tactor on an isomorphic keyboard or screen. Lindeman and Yanagida (2003) tested absolute localization accuracy on the back of participants by using a 3 by 3 array of tactors. They found the accuracy to be high (around 84%) for an inter-tactor distance of 60 mm. However, the threshold of vibrotactile spatial acuity, the minimum detectable distance between tactors, cannot be inferred from their results. Cholewiak et al. (2004) tested absolute localization accuracy on the abdomen by varying the number of tactors in an array in three conditions (12, 8, and 6 tactors). Localization accuracy was highest around the navel and the spine and accuracy increased with fewer tactors and longer distances. The inter-tactor distance was not constant between participants, however, since the tactors were mounted on an elastic band, which the participants wore around the torso and therefore stretched depending on their girth, resulting in different spacing between tactors for each participant. Their results, therefore, do not allow assessment of the minimum detectable inter-tactor distance.

Taken together, even though measurements of absolute localization accuracy can contribute to the improved design of tactile displays, the method is not suitable for determining thresholds for vibrotactile spatial acuity. A number of studies (Eskildsen et al. 1969; van Erp 2005a; Novich and Eagleman 2015) therefore follow a different approach, measuring relative spatial acuity by applying point localization methods (PL, Weinstein 1968). Jones (2011) discusses the two approaches, stating that limitations of the former do not appear to apply to the latter. However, the results of the few studies measuring relative vibrotactile spatial acuity have been inconsistent. Eskildsen et al. (1969) tested the relative position of tactors, using a row of 5 mechanically vibrating (60 Hz) tactors mounted in the back of a dentist’s chair. Seven stimulus distances were tested, ranging from 0 to 60 mm in steps of 10 mm, resulting in mean thresholds of 11 and 10 mm for simultaneous and successive presentation, respectively. Also, van Erp (2005a) tested horizontal spatial acuity using an array of 14 tactors on one occasion and an array of 11 tactors on another (in both cases at 250 Hz) where the distance between the centers of the tactors was 20 mm. Unfortunately, van Erp did not report the absolute accuracy ratio but with a fitting procedure they estimated that the discrimination threshold was between 20 and 30 mm, and approximately 10 mm around the navel and the spine. Van Erp (2005a) also tested vertical acuity, concluding that it was similar to horizontal acuity. Recently, Novich and Eagleman (2015, experiment 2) reported surprisingly low tactile sensitivity for an array of 5 × 2 tactors on their participant’s backs finding that the tactors needed to be at least 60 mm apart for two independent vibrating stimuli to be discriminable at more than 80% correct, regardless of stimulus type (e.g., spatiotemporal “sweeps” versus single vibratory pulses).

### Current goals

Our aim was to systematically investigate the relative spatial acuity on the torso to vibrotactile stimulation with the ultimate goal of using the results to formulate guidelines for inter-tactor spacing when designing tactile displays with vibrotactile actuators. We report the results of 3 experiments where vibrotactile spatial acuity was assessed with “vibro-sponge” devices where 9 tactors (in a 3 by 3 array) were mounted on foam material and strapped to observers’ torsos, and with a tactile vest with 64 tactors in an 8 by 8 array.

## Methods

Three experiments were conducted to systematically investigate the relative vibrotactile spatial acuity of the torso using two types of stimulation devices, a “vibro-sponge” and a tactile vest.^2^ The center-to-center (c/c) distance between the tactors on the vest was fixed to 40 mm in all experiments. The distance between the tactors on the vibro-sponge was gradually decreased from 30 to 13 mm c/c in experiments 1, 2 and 3, respectively, towards the lower limit of possible distance without inter-tactor contact. The general methods used in all experiments are described below.

### Participants

A full within-subjects design would have entailed advance decisions on the minimal tested distance. Since the minimal distance was what we were looking for this was not possible. To compensate for this, each group of participants in the different experiments also performed a task with the tactile vest (see below) that was identical within-experiment, in an effort to assess whether there were any major sensitivity differences between the groups that could account for potential differences in performance on the vibro-sponge tests.

Participants in experiment 1 were 10 (5 F, 5 M) aged between 20 and 38 years (*M* = 28.6 years, SD = 5.3 years). In experiment 2 there were 10 participants (5 F, 5 M) aged from 20 to 31 years (*M* = 24.3 years, SD = 2.9 years) and in experiment 3 there were 10 participants (5 F, 5 M) aged between 22 and 31 (*M* = 26 years, SD = 3.2 years). The participants differed between the three samples with the exception that half of the participants of experiment 2 also participated in experiment 3. All participants were naïve about the purpose of the study and were students or staff at the University of Iceland, and gave written informed consent before participating. All experiments were approved by the National Bioethical Committee of Iceland (VSN-15-107).

### Apparatus

**Fig. 1:**
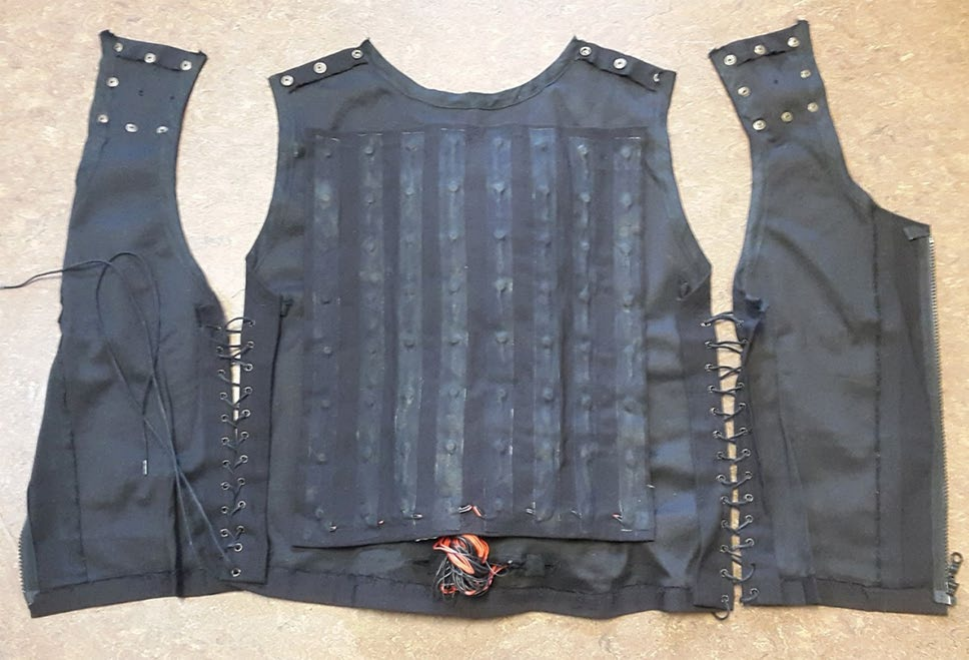
The tactile vest with a 40-mm inter-tactor spacing (used in experiments 1–4)

**Fig. 2:**
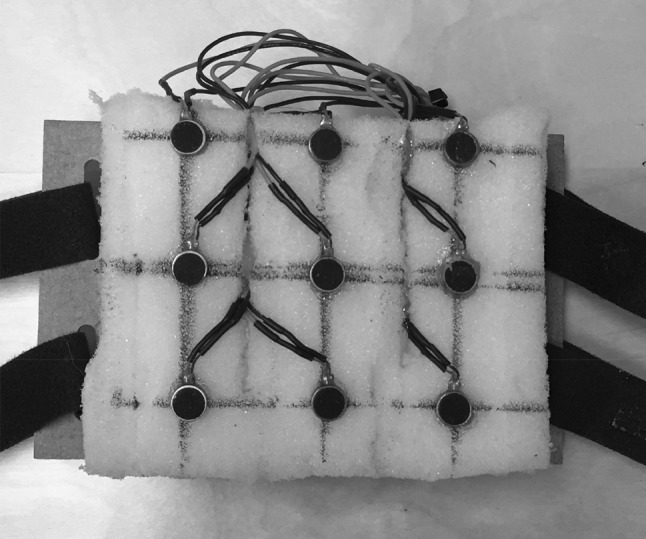
The vibro-sponge with a 30-mm inter-tactor spacing (as used in experiment 1) and similar devices with an inter-tactor distance of 20 and 13 mm were used in experiments 2 and 3, respectively

In all experiments, we used both a tactile vest (40 mm c/c distance, Fig. 1) and a vibro-sponge (30, 20 and 13 mm c/c in experiments 1, 2 and 3, respectively, Fig. 2). The purpose of this arrangement was to assess whether any changes in accuracy with the vibro-sponge reflected individual differences. If similar results are observed for the tactile vest across the groups tested in the different experiments, this supports the conclusion that any inter-tactor distance differences reflect general acuity differences rather than individual differences. Half the participants in each experiment started with the vest and half with the vibro-sponge.

#### Tactors

All devices were equipped with eccentric rotating mass (ERM) tactors (also called coin cell motors), with in-plane vibration (i.e., the vibrations were parallel to the skin). Custom software written in PsychoPy (Peirce 2007, 2009) was used to control stimulus presentation by sending the relevant command through the virtual serial port to a custom-built electronic circuit that controlled the tactors. The diameter of the tactors used on the vibro-sponge was 10 mm and their weight was 0.9 g. The tactors were running on 5 V and the frequency was 183 Hz at full speed (11,000 RPM). The diameter of the tactors used on the tactile vest was 8 mm, otherwise their specifications were similar to those used on the vibro-sponge.

#### Tactile vest

The tactile vest consists of 64 (8 by 8) tactors (8 mm diameter) that are mounted at a horizontal c/c distance of 40 mm, and a vertical c/c distance of 52 mm on the back (see Fig. 1). In experiments 1–3 all the tactors on the vest were used (determined randomly for each trial) but in experiment 4, which served as a control, we used 9 tactors (in a 3 by 3 grid) located at similar locations as the tactors on the vibro-sponge (see below). The aim of experiment 4 was to test whether different tactor numbers between the vest and the vibro-sponge affected performance. The size of the tactile vest was individually adjustable (note that this did not affect intertactile spacing) and participants wore it over their own shirts.

#### Vibro-sponge

The vibro-sponge was used for testing distances smaller than 40 mm c/c in experiments 1–3 since the inter-tactor distance on the tactile vest was not adjustable. The vibro-sponge consists of a 3 by 3 tactor array (see Fig. 2) and was placed centrally on the participants’ back. The c/c distance between the tactors (10 mm diameter) was 30 mm in experiment 1, 20 mm in experiment 2, and 13 mm in experiment 3. As with the vest, the size of the vibro-sponge was individually adjustable and participants wore it over their own shirts.

### Procedure

In all experiments the task was a 3-alternative forced choice (3AFC) task that involved judging whether the second tactor that was activated, was to the left or right of the one activated first or whether it was the same one. Each trial began following a random interval between 1100 and 1700 ms in 100 ms steps. Participants used the left and right arrow keys on a keyboard to judge the location of the second tactor relative to the first one and the space bar if they thought that the second tactor was at the same location as the first. Importantly, participants wore headphones playing white noise during the experiment to mask the sound of the motors,^3^ which could otherwise be a cue. The tactors were turned on for 200 ms with a 50-ms delay between the offset of the first tactor and the onset of the second. The location of the first tactor, and whether the second tactor was to the left, right or the same as the first was randomly determined. The total number of different tactor combinations for the tactile vest was 144 and each combination was repeated 4 times resulting in 576 trials (experiments 1, 2 and 3). The total number of combinations using the 3 by 3 array with the vibro-sponge (and the vest in experiment 4) was 9 and each combination was presented 15 times resulting in 135 trials.

### Statistical analyses

Before analyzing response times (RTs), trials with RTs that deviated more than 3 standard deviations (SDs) from each individual’s mean as well as trials where RTs were less than 100 ms and all trials with incorrect responses were removed. We used R (R Core Team 2015) running in the RStudio environment (RStudio Team 2015) for all analyses. To assess the significance of any effects of distance and horizontal location on accuracy and response times, we used repeated measures ANOVAs (aov; R Core Team 2015). In all experiments there were three possible response types so that chance level was 0.33. One-sample *t* tests were used to assess whether accuracy differed significantly from chance (*t* test; R Core Team 2015) and the Tukey’s honest significant difference test was used for all post hoc comparisons (Tukey HSD; R Core Team 2015). We also measured whether performance would improve with repetition that might reflect practice or habituation.

## Results

### Tactile vest in experiments 1–3

**Fig. 3:**
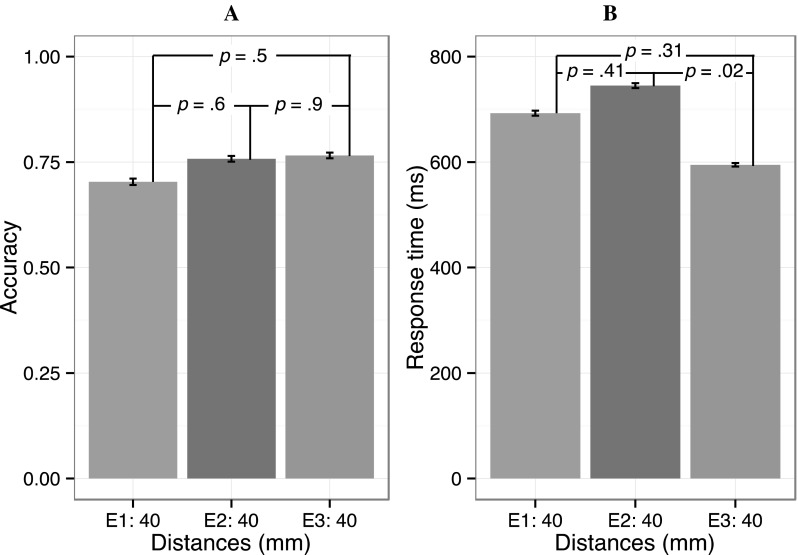
Accuracy and response times for the tactile vest. The figure shows average accuracy (*panel* **a**) and RT (*panel* **b**) for the tactile vest in the 3 experiments. The *error bars* show 2× within-subjects’ SEMs, and the *numbers on the lines* denote the *p* values for post hoc comparisons between these conditions. The accuracy never significantly differed between experiments where the vest was used, while the difference in RT between experiments 2 and 3 was significant

Since the main purpose of using the tactile vest was to assess any differences between the groups and to establish a baseline, we therefore report the results of those tests together in Fig. 3.

The average accuracy for the tactile vest in experiments 1, 2 and 3 was similar for the groups suggesting that individual variation is unlikely to explain any effects of different distances with the vibro-sponge. Average accuracy in experiments 1, 2 and 3 ranged from 0.70 to 0.77, and always significantly differed from chance (see Fig. 3a). Repetitions did not increase accuracy in any of the experiments (all *p*s > 0.2), and there were no differences between the genders (all *p*s > 0.19).

Response times differed significantly between experiments 2 and 3 which might be because half of the participants in experiment 3 had previously participated in experiment 2. None of the other comparisons revealed significant differences (Fig. 3b). If anything, this may suggest that the group in experiment 3 performed slightly better than the others, but this conclusion is premature since accuracy was comparable to accuracy for the groups in experiments 1 and 2. RTs decreased as a function of repetitions in experiments 1, 2 and 3 (see further discussion below). In experiment 1 the average RT in the first block was 751 ms (SD = 261 ms) and in the last block it was 648 ms (SD = 222 ms). In the first block in experiment 2 the average RT was 827 ms (SD = 292 ms) and in the last block it was 671 ms (SD = 210 ms). In experiment 3 the average RT in block 1 was 620 ms (SD = 203 ms) and decreased to 563 ms in block 4 (SD = 166 ms). This decrease in response times with practice may reflect that performance improved with practice for the tactile vest. The most important result is, however, that accuracy was comparable for the groups in the 3 experiments, suggesting that there are little differences in tactile performance between the 3 groups with identical stimuli.

### Vibro-sponge

**Fig. 4:**
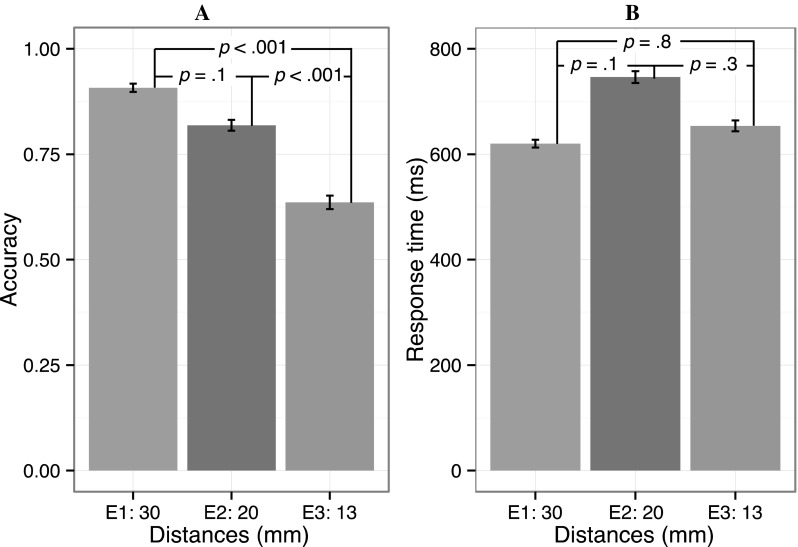
Accuracy and response times for the vibro-sponge. The figure shows the average accuracy (*panel* **a**) and response times (*panel* **b**) for the tactile vest in experiments 1–3 (30, 20 and 13 mm, respectively). The *error bars* show 2× within-subjects’ SEMs and the *numbers on the lines* denote the *p* values for post hoc comparisons between these conditions

Mean accuracy and response times are shown in Fig. 4 for the 3 distances. The tactors on the vibro-sponge were in a 3 by 3 grid (3 columns and 3 rows). Therefore, the tactors were within an area in which accuracy has been reported to be higher than further away from the spine (see e.g., van Erp 2005a). Because of this, generalizing our results to areas further away from the spine should be done with caution. There was no difference in accuracy between the genders (all *p*s > 0.58).

### Experiment 1 (30 mm)

The aim with experiment 1 was to determine the accuracy that can be achieved with an inter-tactor distance of 30 mm (c/c) with the vibro-sponge. Average accuracy was 0.91 (SD = 0.29) and differed significantly from chance (0.33), *t*(9) = 37.91, *p* < 0.001. Performance was close to ceiling, leaving little room for improvement, so the effect of repetitions was not significant [*F*(14,126) = 1.06, *p* = 0.401]. To ensure that there were no artifacts from any potential differences between individual tactors, we also tested whether there were any significant differences between rows or columns. No such effects or interactions were found (all *p*s > 0.24). After removing 130 incorrect responses (8.8% of the data) and 25 outliers (1.8% of the remaining data), average response time was 620 ms (SD = 209 ms). The main effect of repetitions was not significant, *F*(14,126) = 1.25, *p* = 0.248. The main effect of stimulation rows on RTs was significant [*F*(2,18) = 6.95, *p* = 0.006], but a Tukey’s post hoc test showed that there were no significant differences between the different rows (all *p*s > 0.4).

### Experiment 2 (20 mm)

In experiment 2 we tested accuracy with an inter-tactor distance of 20 mm (c/c) with the vibro-sponge. The average accuracy was 0.82 (SD = 0.39) and differed significantly from chance; *t*(9) = 12.73, *p* < 0.001. Accuracy did not change with repetitions; *F*(14,126) = 1.15, *p* = 0.325. Again, we found neither significant main effects nor interactions of stimulation rows and columns on accuracy (all *p*s > 0.06). After removing 245 incorrect responses (18.1% of the data) and 23 outliers (2.1% of the remaining data), average response time was 746 ms (SD = 300 ms). The effect of repetitions on response times was not significant *F*(14,126) = 1.22, *p* = 0.270), and neither stimulation rows nor columns nor the interaction between them affected response times (all *p*s > 0.3).

### Experiment 3 (13 mm)

In experiment 3, we measured accuracy with an inter-tactor distance of 13 mm (c/c), using the vibro-sponge. This is the smallest distance possible when using coin cell motors with a diameter of 10 mm as they would collide if placed closer to one another. Average accuracy was 0.64 (SD = 0.48) and was significantly above chance, *t*(9) = 9.04, *p* < 0.001. Repetitions did not increase accuracy; *F*(14,126) = 0.92, *p* = 0.535. As before, neither main effects of rows and columns on accuracy nor interactions between them were significant (all *p*s > 0.12). After removing 492 incorrect responses (36.4% of the data) and 18 outliers (2.1% of the remaining data), average response time was 654 ms (SD = 240 ms). The response times decreased as a function of repetitions, *F*(14,126) = 1.78, *p* = 0.049. The mean RT in block 1 was 749 ms (SD = 283 ms) and in block 15 it was 603 ms (SD = 207 ms). There were neither significant main effects on RTs of rows and columns nor significant interactions between them (all *p*s > 0.1).

### Comparison of accuracy across experiments

To assess effects of inter-tactor spacing for spatial vibrotactile acuity using the vibro-sponge, and in an attempt to assess tactile acuity for such stimulation, we combined the data from experiments 1, 2 and 3, comparing accuracy and RTs between them. While we realize that the groups are not fully comparable, and comparisons across experiments must carry that caveat, we note, importantly, that the results for the tactile vest revealed no differences between the groups on a comparable task, decreasing the likelihood that group differences explain the patterns. In fact, if anything, the RTs suggest that participants in experiment 3 may have performed slightly better than others with the tactile vest.

To assess effects of c/c distance on accuracy, we compared accuracy rates between experiments 1, 2 and 3 (c/c 30, 20 and 13 mm, respectively). The main effect of experiment was significant [*F*(2,18) = 19.26, *p* < 0.001]. Figure 4 shows the post hoc comparisons. Accuracy was always well above chance (0.33), even with the smallest inter-tactor distance of 13 mm c/c, ranging from 0.66 to 0.91 (all *p* values < 0.001). In sum, the results suggest that the vibrotactile spatial acuity of the torso is equal to or smaller than 13 mm since accuracy is above chance even for this smallest c/c distance. But we also note, importantly, that the accuracy rates significantly decreased (*p* < 0.001) between 20 mm (experiment 2) and 13 mm (experiment 3) and between experiments 1 and 3 (*p* < 0.001). However, there was no significant difference (*p* = 0.122) between the distances of 30 mm (experiment 1) and 20 mm (experiment 2), although the trend was certainly in that direction. This might reflect a ceiling effect for performance. But most importantly, accuracy decreased significantly between the 20 and 13 c/c distances which suggests that at 13 mm distance we are honing in on the absolute threshold. In any case, it is safe to assume that the threshold falls somewhere below 13 mm.

### Comparing the vibro-sponge and the tactile vest

In experiments 2 and 3, accuracy was higher overall for the vibro-sponge than the tactile vest, even though the c/c distance was smaller for the vibro-sponge in both cases. For example, in experiment 1, accuracy was 0.70 for the vest (40 mm c/c) but 0.91 with the vibro-sponge (30 mm c/c). Experiment 2 reveals a similar pattern: the vest with 40 mm (c/c) distance leads to numerically lower accuracy rates (*p* = 0.185) than the sponge with 20 mm (c/c) distance. In experiment 3 accuracy with the vibro-sponge was numerically lower than for the vest (*p* = 0.055), but note that the inter-tactor distance was 40 mm for the vest but only 13 mm for the vibro-sponge. It is likely that various hardware differences between the devices can explain this. The sponge could be worn tighter to the body, and fit more snugly against the back than the vest, and the vest may have caused additional tactile experiences that could add noise during perceptual judgements. But the most important result from comparing the two stimulation devices is that performance was constant between the groups for the tactile vest, while for the same participant groups, performance on the vibro-sponge decreased significantly as the inter-tactor distance decreased. This allows us to be confident that individual differences between the groups do not account for the decreasing accuracy for the vibro-sponges as a function of distance.

### Repetition effects on response times within experiments

While accuracy did not increase throughout experiments (all *p* values > 0.26), response times decreased with increased repetition for the vest (64 tactors; all *p*s < 0.001) but neither for the vibro-sponge (all *p* values > 0.05) nor for the vest when tested with 9 tactors (*p* = 0.224; experiment 4, see below). Stable accuracy and shortened response time do not suggest speed accuracy trade-offs, but rather that participants needed less effort to perform the task as the experiment progressed.

### Effects of c/c distance on response times

As for accuracy, we compared effects of distance on response times between experiments in the combined data set. Differences in RTs as a function of distance were not far from being significant in experiment 1 (*p* = 0.055), but not in the other experiments (all *p*s > 0.1; see Fig. 4). The fact that response times were similar in all experiments shows that speed/accuracy trade-offs cannot account for the accuracy differences by c/c distance for the vibro-sponge between experiments 1, 2 and 3.

### Experiment 4—controlling for the area of possible stimulation on the tactile vest

Experiment 4 was a control experiment to check whether the fact that for the tactile vest the vibration could occur anywhere within the 8 by 8 tactor grid might account for decreased accuracy compared to the vibro-sponge where the stimulation was confined to a 3 by 3 array. We compared performance on the vest when the stimulation could be anywhere on the grid during the experiment and when the possible locus of stimulation was confined to a 3 by 3 grid (at similar locations on participant’s backs as for the vibro-sponge) throughout the experiment. Participants in this control experiment were the same as in experiment 3 and we therefore used data from experiment 3 (tactile vest, c/c 40 mm). In experiment 3 the average accuracy was 0.76 (SD = 0.42) and in experiment 4 it was 0.75 (SD = 0.43). The difference in accuracy was not significant [*F*(1,9) = 0.09, *p* = 0.772]. The average RT in experiment 3 was 595 ms (SD = 184 ms) and in experiment 4 it was 649 ms (SD = 289 ms). The difference (54 ms) was not significant [*F*(1,9) = 1.25, *p* = 0.293]. Repetitions neither increased accuracy (*p* = 0.791) nor shortened RTs (*p* = 0.127). Overall, the results show that performance differences between the tactile vest and the vibro-sponges cannot be explained by differences in stimulation area.

## Discussion

While a lot is known about the spatial acuity of tactile perception when it comes to pressure, far less is known about sensitivity to vibrating stimuli. Here we systematically investigated the relative spatial acuity of the torso’s skin to vibrotactile stimulation. Our aim was firstly to gain better understanding of tactile sensitivity to vibration and secondly to assist in formulating guidelines for inter-tactor spacing during the design of tactile displays that use vibrotactile stimulation. We conducted 3 experiments involving two types of stimulation devices mounted with coin cell motors. We gradually decreased the center-to-center inter-tactor distance from 30 to 13 mm c/c.

Accuracy in all experiments was well above chance suggesting that the spatial acuity of the torso’s skin is lower than 13 mm c/c. Accuracy nevertheless dropped significantly for the 13 mm distance compared to the longer distances. This result is in line with Eskildsen et al. (1969), who found a two-point threshold of 10 mm for successive stimulus presentation on the torso. Even though the spatial acuity in our study appears higher than in Van Erp (2005a), who estimated that the spatial acuity across the torso was 20–30 mm, the difference may partly reflect the location of the vibro-sponge in our experiment. Since the vibro-sponge was placed centrally on the back, mainly covering the spine region, the tactors stimulated an area for which accuracy has been reported to be higher than further away from the spine (Van Erp 2005a). Hence, the threshold found in our study is valid for the center area of the back and generalizing it to lateral areas should be done with caution.

Conversely, our results are discrepant with those of Novich and Eagleman (2015) who found spatial acuity to vibrotactile stimulation to be only 60 mm with a tactile vest. It is interesting to compare their results with our results for the tactile vest (Fig. 3), since the spatial acuity was significantly lower than for the vibro-sponge, which probably involves more direct stimulation than the tactile vest. The inter-tactor distance was 40 mm for our tactile vest, but accuracy levels hovered between 70 and 80%, way above chance level. Note also that the task in Novich and Eagleman was not fully comparable to ours, but it is unlikely that superficial task differences can explain the difference in accuracy estimates.

The main practical conclusion that can be drawn regarding the design of tactile devices in the torso area, is that coin cell tactors of 10 mm diameter can be placed as close as possible (13 mm c/c), for above chance performance, although our results also suggest that performance drops a bit at this point. But the person wearing the device will still be able perceive the tactors individually, which is a key requirement for successfully conveying information using a tactile language. Yet another consideration is that above chance performance may not be a particularly ambitious goal for conveying information. Our aim was not to determine accuracy levels necessary for any particular device, so any such criteria must be set by the required resolution for a particular device.

Our hope is that our findings may help with designing vibrotactile equipment in general and sensory substitution devices more generally. Knowledge of spatial acuity thresholds is important for optimizing displays to fit the perceptual characteristics of the torso. The specific interest in the torso has increased, following recent successes in the application of vibrotactile torso displays in orientation and navigation tasks in various contexts (Van Erp et al. 2004). By presenting a spatio-temporal pattern, these displays can indicate the direction of drift in a helicopter hover task or the direction of the next waypoint in a navigation task. The more tactors that can be placed within a tactile display, the more information can be conveyed without the need to additionally encode information by deploying the dimension of time. Therefore, determining spatial acuity thresholds should lead to more efficient tactile applications.

When determining the vibrotactile spatial acuity of the torso’s skin, some possible confounding variables have to be considered, which may explain the inconsistent findings. Hence, in the following section, we outline and discuss some of these issues.

One factor that may influence tactile spatial acuity is the number of tactors and the distance between them, or in other words, the size of stimulation area, which can vary greatly between studies. Eskildsen et al. (1969) tested arrays of 5 × 1, van Erp (2005a) tested arrays of 14 × 1 and 11 × 1 and Van Erp et al. (2005b) tested 8 tactors. Using an array of 3 by 3 tactors with c/c 60 mm (horizontal and vertical, Lindeman and Yanagida (2003) reported absolute spatial accuracy of 84%. Jones and Ray (2008) used an array of 4 by 4 tactors with 60 mm c/c, horizontal and 40 mm vertical, to test absolute spatial localization and found the average accuracy across all tactors to be 59%. Further analyses of the data revealed that horizontal accuracy was 87% but 68% vertically. Accuracy by distance (c/c) was very similar in Lindeman and Yanagida (2003) and Jones and Ray (2008) although the number of tactors differed considerably between these experiments (9 versus 16, respectively). Cholewiak et al. (2004) concluded that the most important factor for localization accuracy is inter-tactor distance. Their results show that the effect of number of tactors on localization is ambiguous. Our results on relative spatial acuity with point localization suggest that decreasing the size of the area of vibrotactile stimulation does not influence thresholds for vibrotactile spatial acuity as there is no significant drop in accuracy when the same device (the tactile vest) is used with a 3 by 3 or an 8 by 8 tactor array. The tactile two-point threshold could also vary by the tested direction. In our study, only the horizontal axis was tested, but Van Erp (2005a) did not find any difference by direction. However, further studies systematically investigating the influence of stimulation area size, as well as direction, and comparing the results for absolute and relative spatial acuity are necessary.

The design of tactile devices can cause variability when spatial acuity is measured. We found that the vest with 40 mm (c/c) distance led to significantly lower accuracy rates than the sponge with 30 mm (c/c) distance. The main difference in the design is that the tactors on the vibro-sponge are mounted on soft foam and are not covered with fabric. The foam minimizes the distribution of the vibration from the tactors probably resulting in more fine-tuned localization of the vibrations. Van Erp (2005a) attached the motors directly to the skin using thin double-sided adhesive tape, whereas the participants in our study wore their own shirts under the tactile devices. This calls for further investigation of the influence of design of tactile devices on spatial acuity.

We should note that the spatially static stimulation we used here may underestimate thresholds. Vierck and Jones (1969) demonstrated that the discrimination of the size of rounded stimuli (discs) is about ten times better than for point stimuli and Jones and Vierck (1973) found that the discrimination of line lengths was about two times lower than for 2PL, suggesting that patterns could provide more information than point stimulation. Gibson (1962) found large differences between passive tactile perception of a stationary versus moving stimulus. When a ‘cookie-cutter’ was pushed onto participants’ palms while remaining otherwise stationary, identification rates of its pattern were just under 50% percent, but if the cutter was pushed around in the observers’ palm, recognition accuracy became about 95% (see also Novich and Eagleman 2015). Moving patterns may therefore increase the actual resolution.

Furthermore, the chosen paradigm can influence spatial thresholds. When comparing PL and 2PL methods, Weinstein (1968) found that spatial tactile acuity with the two-point threshold was 2–4 times lower than with point localization although both thresholds were highly correlated (e.g., thresholds for the back were ca. 40 mm for the PL and 10 mm for the 2PT). However, the former method, 2PL, cannot be applied with tactors, as it requires comparing the conditions of running either one tactor or two tactors to find out if the two-tactor condition is perceived as one. Vibrotactile simulation involves frequencies with particular phase, which leads to a noticeable phase difference as soon as two vibrotactile tactors with different phases run at the same time. These phase differences are clear indicators of simultaneously running tactors, which participants can base their decision on. We therefore applied the point localization paradigm by presenting two successive stimuli instead of the 2-point-threshold approach. Additionally, it is important to note that results on relative spatial acuity as in this study are not directly comparable to measurements of absolute spatial acuity. The ability to localize a point of vibrotactile stimulation on the back (absolute) does not appear to reflect limitations with relative spatial acuity (Jones 2011).

Finally, the high variance in findings on spatial acuity may stem from different tactor types. The complex nature of vibro-tactile stimulation renders comparisons of studies investigating vibro-tactile spatial acuity thresholds difficult. The physical characteristics of vibrotactile signals itself can vary by amplitude and frequency although there is as fixed relationship between frequency and amplitude of ERM tactors (Jones 2011; Precision Microdrives 2017) that are commonly used and we used in our experiments. Comparisons across studies using different tactors are further limited by interactions between vibro-tactile signal dimensions. For instance, the frequency and amplitude of vibration are not orthogonal, since changes in the frequency of a vibrotactile signal can affect their perceived amplitude (Bolanowski et al. 1994; Morley and Rowe 1990) and vice versa (Verrillo et al. 1969). Spatial acuity thresholds measured with one tactor type should be cautiously generalized to other tactor types. In future work, we will therefore assess vibrotactile spatial acuity with different tactor types.

## Footnotes

1 A tactor, short for tactile vibrator, is a motor that creates vibrations.

2 We ran a control experiment (Experiment 4) to compare performance with the vest with 8 by 8 versus 3 by 3 tactors. Those results are discussed at the end of the “Results” section.

3 Before running the main experiments, we ran a pilot experiment to find the white noise level that adequately masked the sound from the tactors.
